# EcoQBNs: First Application of Ecological Modeling with Quantum Bayesian Networks

**DOI:** 10.3390/e23040441

**Published:** 2021-04-09

**Authors:** Bruce G. Marcot

**Affiliations:** Forest Service, Pacific Northwest Research Station, Portland, OR 97208, USA; bruce.marcot@usda.gov

**Keywords:** quantum Bayesian networks, EcoQBN, intransitive networks, noncommutative networks, quantum amplification, ecology

## Abstract

A recent advancement in modeling was the development of quantum Bayesian networks (QBNs). QBNs generally differ from BNs by substituting traditional Bayes calculus in probability tables with the quantum amplification wave functions. QBNs can solve a variety of problems which are unsolvable by, or are too complex for, traditional BNs. These include problems with feedback loops and temporal expansions; problems with non-commutative dependencies in which the order of the specification of priors affects the posterior outcomes; problems with intransitive dependencies constituting the circular dominance of the outcomes; problems in which the input variables can affect each other, even if they are not causally linked (entanglement); problems in which there may be >1 dominant probability outcome dependent on small variations in inputs (superpositioning); and problems in which the outcomes are nonintuitive and defy traditional probability calculus (Parrondo’s paradox and the violation of the Sure Thing Principle). I present simple examples of these situations illustrating problems in prediction and diagnosis, and I demonstrate how BN solutions are infeasible, or at best require overly-complex latent variable structures. I then argue that many problems in ecology and evolution can be better depicted with ecological QBN (EcoQBN) modeling. The situations that fit these kinds of problems include noncommutative and intransitive ecosystems responding to suites of disturbance regimes with no specific or single climax condition, or that respond differently depending on the specific sequence of the disturbances (priors). Case examples are presented on the evaluation of habitat conditions for a bat species, representing state-transition models of a boreal forest under disturbance, and the entrainment of auditory signals among organisms. I argue that many current ecological analysis structures—such as state-and-transition models, predator–prey dynamics, the evolution of symbiotic relationships, ecological disturbance models, and much more—could greatly benefit from a QBN approach. I conclude by presenting EcoQBNs as a nascent field needing the further development of the quantum mathematical structures and, eventually, adjuncts to existing BN modeling shells or entirely new software programs to facilitate model development and application.

## 1. Introduction

The use of Bayesian networks (BN) has become a popular tool for ecological modelling in recent years. BNs are directed acyclic graphs which link variables by probabilities based on first-order Markov relations. The uses of BNs are many, including the analysis of the impacts of storm events on coastal environments [1], the assessment of ecosystem services and avalanche protection [2], the applications of informing decision-making under uncertainty [3], and much more. The advantages of BNs over traditional, frequentist statistical approaches are in their flexibility to handle multiple kinds of data, capacity to integrate expert-elicited knowledge, robustness to missing data, availability of machine-learning algorithms for the structuring and parameterizing of the models, and especially in their explicit representation of uncertainty and its propagation in calculations of probability outcomes [4,5,6].

However, there are problems central to social psychology, logic, beliefs, and even ecology which defy adequate representation by classical Bayesian analysis and BN model structures. For one, predicaments arise on what to do when a situation becomes a problem in which the dominant probability outcomes are nonintuitive and defy traditional probability calculus. Much of ecosystem science is fraught with stochastic relationships and high uncertainty, rendering accurate estimations of outcome probabilities quite difficult at best. This article explores such problems in general and offers a fresh approach to BN modeling in ecology which can help solve such difficulties.

A recent advancement in modeling is the development of quantum Bayesian networks (QBNs). Here, I present a novel introduction to areas of ecological description and analysis, introducing their use as Ecological Quantum Bayesian Networks (EcoQBNs). I first describe the form of problems which are not amenable to traditional BN modeling. I then explain QBN structures and approaches, present three case examples of EcoQBNs pertaining to ecological problems, and conclude with suggested next steps for the advancement of this new field of ecological modeling. My intended audience for this introduction is quantitative ecologists.

## 2. Methods

### 2.1. Quantum Bayesian Networks

In quantum mechanics, a wave function is a mathematical construct which embodies the dynamics and uncertainties of a system, including the probability amplitudes of the various states of the system. A QBN—quantum Bayesian network—is essentially a probability network in which the traditional Bayesian probability calculus is replaced by quantum probability amplitudes.

Goyal and Knuth [7] showed that quantum theory and probability theory are not incompatible, although classical probability theory cannot depict the fuller suite of quantum system dynamics and interactions. QBNs were introduced by Tucci [8] as a means of calculating quantum mechanical conditional probabilities in the field of magnetophysics. The theory and application of QBNs is published mostly in literature pertaining to problems in physics [9,10], and in social psychology and cognitive science related to irrational decision-making [11,12,13,14,15]. In general, QBNs can be used to describe and solve a variety of problems in probability which are unsolvable by, or too complex for, traditional BNs, essentially where the outcomes are nonintuitive and defy traditional probability calculus (Table 1).

### 2.2. Problems Addressed with Quantum Bayesian Networks

Outside the realm of quantum physics, QBNs have been used to describe a variety of human cognitive situations which do not conform to calculi used in traditional probability theory or Bayesian statistics, including the use of Markovian processes and Boolean algebra [16,17]. It is this set of applications which spurred this current exploration into their use in ecological modeling.

#### 2.2.1. Problems of Illogic and Irrationality

In psychology, QBNs are applied to address the situation in which the beliefs of humans do not follow the rules of Boolean logic or classical probability theory. One example in social psychology is with the so-called Ellsberg paradox, which is also referred to as the heuristic of insufficient reason, which describes how people adhere to familiar behaviors and make decisions that may not yield the greatest benefit despite clear evidence of their suboptimal outcomes. The logical relations inherent in a classical probability approach would fail to adequately represent the illogic of insufficient reason. Alternatively, Asano et al. [18], al-Nowaihi and Dhami [19], and Moreira et al. [20] developed quantum probability decision models that agreed well with observed behaviors of this type.

Other human behaviors which violate classical probability analysis that have been modeled with QBNs include how people tend to hold conflicting beliefs at the same time, which has been analogized to quantum wave functions with interference patterns [21,22]. In classical Bayesian analysis, such behaviors would constitute a situation in which all of the probabilities *P* of all potential states (beliefs) *i* sum to >100% ($\underset{i}{\sum }P_{i}\;>\;1.0$), which is a violation of the Law of Total Probability in traditional probability theory, in which all probabilities must sum to 100% [16]. In QBNs, beliefs (e.g., probabilities of BN states) become overlapping, non-mutually-exclusive wave functions, with interference patterns. That is, different, contrasting beliefs (states) can be held at the same time, but they can also interfere with one another until some empirical outcome or experience occurs which then resolves the belief wave functions down to classical probability outcomes, or to just one state (one belief).

Additional situations in psychology which are amenable to QBN modeling include other irrational behaviors such as with placing bets in which, if a bet is won it is played again, if a bet is lost it is still played again, but if it is not told whether it is a win or a loss, the bet is not played again [22]. This type of behavior is referred to as violating the Sure Thing Principle, which describes otherwise logically self-consistent and rational decisions, and has been modeled with QBNs [13,23,24]. A similar irrational behavior is to continue adopting a losing strategy which eventually yields a winning strategy, which collectively exceeds the total probability (*p* > 1.0) of all outcomes. This situation is referred to as Parrondo’s paradox, which defies traditional probabilistic decision modeling, but also has been represented with QBNs [24,25,26]. In general, such nonintuitive human behaviors which violate rules of classical probability theory tend to result in part because people largely cannot mentally process large amounts of data [22].

#### 2.2.2. General Problems Defying Traditional Probability Calculus

As suggested above, QBNs can be applied to several general categories of problems which are not amenable to representation or solution by traditional probability calculus, which will be explored further below in the context of ecological modelling. These problem categories include systems with feedback loops and temporal dependencies among variables [27], which a traditional BN approach could model only with complex temporal replication and the expansion of the network (e.g., References [6,28]).

Another category is systems with non-commutative dependencies in which the order of the specification of the priors affects the posterior outcomes. Traditional BNs do not specify the cardinal sequence with which the states are specified in parent nodes; the outcome is the same regardless of the sequence, as BNs essentially consist of first-order Markov processes for each link, and direct parent inputs to a variable (node) are considered independent unless they themselves are linked. In traditional Bayes calculus, this means that if A and B are independent variables affecting some outcome C, such that A → C ← B, then *P* (C|A,B) = *P* (C|B,A), in which the sequence of the specification of the values of A and B does not matter. In a noncommutative system in which the sequence does matter, however, these equalities do not hold.

Another category is problems with intransitive dependencies constituting a circular dominance of outcomes. The transitive property says that if A > B and B > C, then A > C. This holds in a traditional BN in which, in traditional Bayes calculus, assuming A, B, C are causally linked in a first-order Markov chain A ← B ← C, this means that if *P* (A|B) > *P* (B|C), then *P* (A|B) > *P* (A|C). In a social context, this general concept means that if person 1 is friends with person 2, and person 2 is friends with person 3, it says that person 1 is friends with person 3. Such seems to be the situation with social ‘friends’, such as on popular social media. But this may not necessarily hold in real life, in which one might not wish to be friends with the friend of a friend, thus conjuring a non-transitive situation. In an intransitive system, A > B, B > C, but C > A.

More esoteric categories in which traditional BN modeling can fail include those in which input variables can affect each other even if they are not causally linked, which is described in quantum mechanics as entanglement; those in which there may be >1 dominant quantum probability outcome (each *P* > 0.5) as noted above, described as superpositioning; those in which small variations in inputs can drastically affect results, described in chaos theory as the butterfly effect; those in which a variable can have a great effect on some outcome even though it may be only distantly linked, or even occurring in a separate network, referred to as quantum tunneling and nonlocality; and those in which the basic causal structure and parameters are fixed, but different trials (model runs), based on the same inputs, can yield different stochastic outcomes [29].

Using QBNs for problems in ecological modeling pertains to identifying ‘quantum-like’ analogues from concepts and calculations in quantum mechanics which are useful for describing and solving otherwise intractable ecological problems. ’Quantum-like’ implies that quantum phenomena strictly do not scale up to macro dimensions, although studies in ‘quantum biology’ suggest a degree of scaling to some phenomena in photosynthesis, avian magnetoreception, evolution, and some other biological phenomena [30,31,32].

### 2.3. The Quantum Math of QBNs

QBNs are structured and solved using mathematical structures borrowed from quantum mechanics, as in the following example. Consider a simple network with two binary (true-false, *t*-*f*) variables, *A* and *B*. Their conjunction is represented in a traditional discrete Bayesian network by a 2 × 2 conditional probability table with entries:

$$\left[\begin{array}{cc} A_{t}|B_{t} & A_{t}|B_{f}\\ A_{f}|B_{t} & A_{f}|B_{f} \end{array}\right]$$

In quantum probability theory, *A* and *B* are represented by their Hermitian operators:

$$A=a_{t}Pa_{t}+a_{f}Pa_{f}$$

$$B=b_{t}Qb_{t}+b_{f}Qb_{f}$$

in which *a_i_* and *b_i_* are real eigenvalues of the probability table, representing physical variables, and *Pa_i_* and *Qb_i_* are projections onto the corresponding eigen sub-spaces. Hermitian operators are used in quantum mechanics to represent >1 overlapping state of a system.

Then, the probability of a specific value *a_t_* is given by Born’s Rule [29]:

$$P\;\left(A=t\right)=<Pa_{t}\psi |\psi >=\|Pa_{t}\psi {\|}^{2}$$

in which ψ is the unit length state vector reflecting knowledge about the variable, also referred to as the wave function. The above formula also uses the linear algebra ‘bra-ket’ notation to denote how a linear function, the ‘bra’ 〈f|, defines a vector space, the ‘ket’ |v 〉 , which represents quantum states. The probability $P\;(B_{f}|A_{t})$, being the (1,2) entry in the above conditional probability table, is calculated as the conditional state vector $\psi_{at}$ by applying Born’s rule:

$$P\left(B_{f}|A_{t}\right)=\langle Qb_{f}\psi a_{t}|\psi a_{t}\rangle \;=\|Qb_{f}\psi a_{t}{\|}^{2}$$

This is a quantum conditional probability. Note that Born’s Rule is used to map quantum amplitudes to classical probabilities. Completing all other members of the 2 × 2 contingency table in this way completes and populates the matrix of all quantum conditional probabilities. This approach will be used below in an example of the calculation of the habitat conditions for a bat species.

### 2.4. Hilbert Space Representation of States

**Figure 1:**
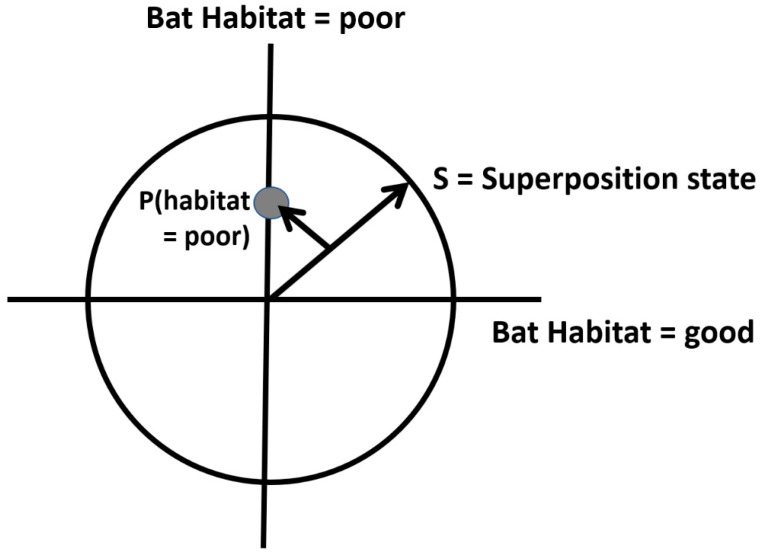
Hilbert state-space representation of two habitat conditions for a bat species (see text). *S* is the superposition condition representing both states simultaneously, and the smaller arrow *P* (habitat = poor) is the projection, as used in quantum mechanics, onto the y axis to derive the (normalized) probability of the habitat being poor (see Supplementary Material S1 for calculations).

Quantum probabilities of alternative states are represented as projections from a unitary vector in what are called Hilbert spaces, in which orthogonal axes represent the alternative state conditions. In a simple example, habitat conditions may be either good or poor for a wildlife species, and there may be some uncertainty as to which is the case, such that the projection defines a point denoting the probabilities of each state (Figure 1). This is analogous to how a BN would calculate the posterior probability values for each state. A key difference is when the events (habitat states) in a quantum Hilbert space are defined by a superposition state represented by the state vector made up of all events (math described in Supplementary Material S1). The state revisions are then calculated by again projecting *S* onto the subspace state, then normalizing the projection so that the resulting vector is of unit length; this gives the probability of that specific state.

### 2.5. Path Trajectories

Path trajectories in ecology can represent such phenomena as the state-transition development of vegetation communities, such as under disturbance and ecological succession (explored below); secondary effects of herbivory on vegetation and the habitat conditions of other species [33]; and other phenomena such as trophic-cascade interactions among organisms and resources for other species, such as how predators can mediate a plant community structure through the control of herbivore populations [34].

A traditional probability representation of a three-step path trajectory A → B → C is calculated as:

P (A→B→C)=P (A)⋅P (B|A)⋅P (C|B)

In a quantum path model, in which *ψ* is the complex probability amplitude (a complex number function which describes the behavior of a system) [22],

(1) $$P\left(A\to B\to C\right)={\left|\psi_{A}\right|}^{2}\cdot {\left|\psi_{B|A}\right|}^{2}\cdot {\left|\psi_{C|B}\right|}^{2}$$

## 3. Ecological Quantum Bayesian Networks in Ecology

The structure of QBNs as described above can be meaningfully extended to many kinds of problems in ecological modeling with EcoQBNs. I first describe the general kinds of ecological problems which can be addressed with an EcoQBN approach, and then I present three diverse examples of potential applications.

### 3.1. Ecological Problems Framed by an EcoQBN Approach

At least four general categories of ecological situations can be modeled with EcoQBNs which would otherwise fit poorly or not at all within traditional statistical and probabilistic frameworks: (1) intransitive ecosystems responding to suites of disturbance regimes with no specific or single dominant or climax condition; (2) noncommutative ecosystems which respond differently depending on the specific sequence of the disturbances (priors); (3) self-organizing systems with mutual-conditioning feedbacks; and (4) systems involving entrainment and the synchronization of signals.

#### 3.1.1. Intransitive and Hyperintransitive Systems

Many ecological systems are inherently intransitive, e.g., with a circular succession of conditions which return to the initial stage, with no one stage dominating. When mapped onto a network, intransitive systems ultimately include a feedback cycle and complete a circuit. Intransitive ecological systems include seasonal or annual breeding cycles, repeat population cycles of numerical response, ecosystems incurring repeat natural or anthropogenic disturbance events amidst successional development, and others. In some systems, one element, such as a particular type of disturbance, can play multiple counterfactual roles; when represented in a planar network graph of the system, at least one intersection occurs across some other relationship, and forms what can be termed ‘hyperintransitivity’ (in which representing the interactions in the network without intersections entails a higher-dimensional depiction). This is essentially a problem in intersection graph theory [35]. In general, global solutions to intransitive or hyperintransitive BNs are infeasible, or at best require overly-complex latent variable structures which might still not specify the conditions for general traditional solutions.

Examples of ecological systems with potentially intransitive circuits include some predator–prey cycles (e.g., lynx–hare [36]), herbivore–vegetation cycles [37], density-dependent limitations on population growth [38], Allee effects [39], and more. Circuits with feedback loops are awkward, at best, to depict in static BN models, and are sometimes solvable via the so-called time-expansion of the network, as with dynamic BNs (DBNs [6,40]).

**Figure 2:**
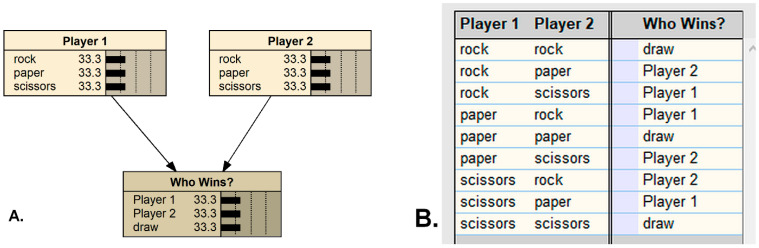
The two-player simultaneous-play game of Roshambo, as represented by a Bayesian network (**A**), with the conditional probability table predicting the outcome (**B**), as programmed in Netica (Norsys Inc., Vancouver, Canada, www.norsys.com, accessed on 7 April 2021).

A classic, non-ecological example of modeling an intransitive system in QBNs is with the well-known two-person game of roshambo, also called rock-paper-scissors. The game is intransitive because there is no strategy which wins all possible games; any play can be a losing move. The BN model for roshambo is simple (Figure 2A), requiring two inputs and one output. However, the strategies leading to a specific result cannot be determined without also specifying one of the player’s moves, nor does specifying only one player’s move determine the outcome without knowing the other player’s move. In general, specifying a state in any one of the three nodes does not solve the rest of the network, or even provide any information on the probabilities of the potential outcomes (Figure 2B). This situation can be referred to as ‘solution-incomplete’, a particular and peculiar feature of intransitive BNs. Also note that the particular sequence in which the inputs are specified, in this game space, determines the outcome, assuming that the players are competing and viewing the others’ move first (although roshambo is a simultaneous-play game); this is noncommutativity, and has implications—discussed below—for how species may interact within evolutionary situations. The only way to depict this fully in a traditional BN model is with latent variables specifying all combinations or sequences of reveals with their specific game outcomes.

The intransitive structure of roshambo has been likened to persistent cycles of species competition in ecological communities, as competitive advantages get traded off among species with no clear winner [41,42]; to the development and stability of species diversity [43,44]; and to the development of high social efficiency in human decision-making [45]. In ecological contexts, Sakai et al. [46] specifically used the Hilbert transform (a mathematical technique for deriving phase amplitudes) to represent an intransitive three-period stable cycle of ‘masting’ in tree crops, and they further [47] described how a coffee agroecosystem can constitute a stable dynamic, intransitive situation with the interactions of three elements of an insect pest, parasitoid, and predator. Further, a prime example of an intransitive ecological system was described by Yule and Burns [48] in New Zealand, who revealed an unexpected additive adverse impact on a native tree species from the reintroducing an insectivore parrot thought to control an herbivorous moth which damages that tree species. However, none of these ecological examples used QBNs per se, although their problems could be reformulated for an EcoQBN analysis.

#### 3.1.2. Noncommutative Systems

As mentioned above, roshambo and its ecological analogues are characterized by the condition of noncommutativity, in which the outcomes are determined by the order in which the inputs are specified (players and species make their moves). BNs are commutative, and the sequence by which input variables (priors) are specified has no bearing on the outcome. To represent noncommutative properties in a BN would entail what could be an exponential expansion of all possible sequences of the specification of the values of the priors, with massive increases in the resulting conditional probability tables in multiple outcome nodes. Instead, a QBN can far more efficiently represent all such possible sequences than can a traditional BN.

An example of a noncommutative ecological system is the response of vegetation conditions to the specific sequence of various disturbance events. This can be denoted in state-transition models, and is discussed further below with an example.

#### 3.1.3. Self-Organizing and Mutually-Conditioning Systems

A third category of systems amenable to the EcoQBN framework are those which are self-organizing with mutually-conditioning arguments. Mutual conditioning refers to mutual causality which, in some systems, can be represented with feedback loops—as discussed above—in which the cause and effect parameters are interchangeable and can be observer-dependent [49]. Feedback loops render elements in a system to become entangled and to mutually conditional on each other, even if there is a final, temporal stability or resolution to the feedbacks. An example of an ecological system with positive feedback is how loss of Arctic sea ice lowers albedo (sunlight reflectance), causing the increased warming of the exposed, darker ocean, which accelerates further loss of sea ice. Other examples include the accelerating movement of tidal glaciers in Greenland with regional warming causing ice-melt to reduce friction, the channeling of the incised river meanders from increased runoff, and other situations.

Mutual causality in biological systems can result in situations of mutual advantage, such as the evolution of mutualistic symbiotic relationships. It could also result in populations with balanced polymorphism (>1 phenotype). Examples of polymorphisms include colonies of the shorebird Steward Island Shag (*Leucocarbo chalconotus*) in New Zealand, which contain both a bronze color morph which is all dark, and a pied color morph which is black and white (but which may be separate species [50]); color morphs of red-backed salamanders (*Plethodon cinereus*) in the U.S. [51]; and gold and dark forms of Midas cichlid fishes (*Amphilophus citrinellus*) in Nicaragua [52].

Mutual causality, with positive feedbacks of increasing amplitudes or negative feedbacks with dampening amplitudes, can be represented efficiently by quantum interference patterns among wave functions. This can result in periodic cycles of states if the causal structure is stable, or as aperiodic eruptions or chaotic behavior if the direction and intensity of feedbacks shift over time. Depending on the structure and dynamics of the system, such cycles and behaviors could be modeled using traditional probability calculus in BNs, although the BNs would themselves become complicated, time-replicated expansions in order to retain an acyclic structure. Furthermore, just because two (or more) repeating events seem synchronized is inadequate evidence that they are causally connected (directly or even indirectly through a latent variable), signaling that caution needs to be taken against false patterns, pseudoentrainments, and pseudosynchronicities.

#### 3.1.4. Systems with Signal Entrainment

A final type of problem discussed here which EcoQBNs can address is systems with signal entrainment, which are systems with elements which interact and respond so as to in some way become coordinated. Signal entrainment—a form of mutual conditioning—occurs in a variety of ecological situations (discussed in examples further below) as two main categories: (1) systems with signal entrainment but not signal simultaneity, which are those with temporal or spatial offsets between the elements, with examples of duetting in birds [53] and predator–prey cycles (noted further above); and (2) systems with signal entrainment and signal simultaneity (true synchronicity), with examples of fireflies flashing, field crickets chirping, and birds within colonies calling in unison [54,55,56].

### 3.2. Examples of EcoQBN Situations

The following are three examples of ecological situations lending themselves to an EcoQBN modeling approach, which otherwise defy traditional BN modeling solutions: habitat selection by a species of bat, transitions of terrestrial ecosystem conditions under disturbance regimes, and signal entrainment in birds and insects.

#### 3.2.1. Example 1: The Habitat of the Yellow-Winged Bat

**Figure 3:**
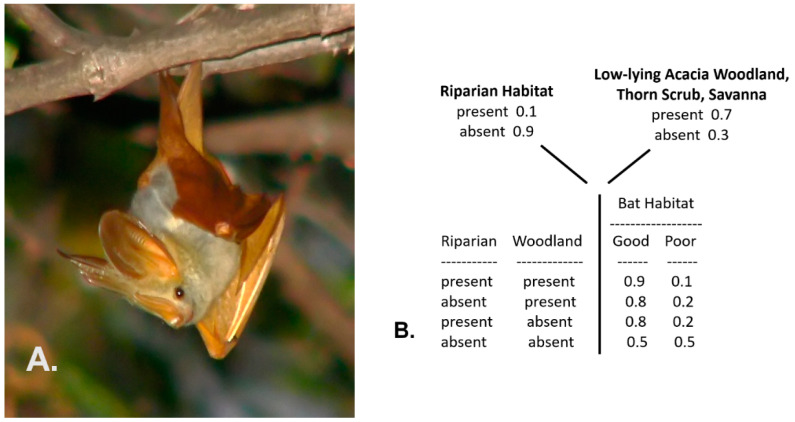
Example of the yellow-winged bat (*Lavia frons*) found in tropical sub-Sahara Africa (**A**), and the probability (frequency) distributions of two environments, combined to denote the conditional probability of the habitat quality for the species (**B**).

This first case follows an example from Trueblood et al. [57], pertaining here to the depiction of the habitat conditions of the yellow-winged bat (*Lavia frons*), a mostly solitary species (Figure 3A) found in sub-Saharan tropical Africa. Yellow-winged bats typically favor narrow forest gallery riparian conditions within low-lying acacia woodlands, thorn scrub, or savanna environments [58]. Thus, a simple conditional probability table can depict their habitat values (probabilities of suitable habitat), as shown in Figure 3B (the values specified in the table are hypothetical and based on my personal observations of the species in east Africa, as no formal probability model has been developed for this species). This table is the basis for a simple traditional Bayesian network.

The bat habitat conditions can also be represented by a Hilbert space, which is a vector space of complex numbers which offers the structure of an inner product to enable the measurement of angles and lengths. As above [Equation (1)], ψ represents the probability amplitude of the wave function, and θ is the shift (translation) of the wave function, the degree of shift being determined by the observed conditions. Further metrics of the amplitude of the wave function are specified by:

$$e^{i\theta }=\mathrm{phase}\;\mathrm{of}\;\mathrm{the}\;\mathrm{amplitude}$$

$$\frac{e^{i\theta }}{\sqrt{2}}=\mathrm{probability}\;\mathrm{amplitude}$$

Note, importantly, that the square of the probability amplitude is the classical probability value.

The entries for the quantum conditional probability table are calculated as follows, presented here as the general framework for the EcoQBN model of this species. The bat habitat conditions *H_good_* and *H_poor_* are defined by a superposition state *S* (a state vector), which is comprised of both of the H states:

$$S\;=\;\frac{e^{i\theta good}}{\sqrt{2}}H_{good}\;+\;\;\frac{e^{i\theta poor}}{\sqrt{2}}H_{poor}$$

The two habitat conditions, good and poor, can be graphically represented in a Hilbert space, as shown in Figure 1. In this graphical depiction of uncertainty, the habitat can be in both good and poor states simultaneously, to varying degrees (probabilities).

Next, in order to derive the classical condition probability values, we square the quantum probability amplitude. This is achieved by multiplying the quantum amplitude with its complex conjugate (also see Supplementary Material S1). For the probability of a poor habitat, *P (H_poor_)*, the superposition state *S* is projected into the subspace representing the observed event (state), in this case shown as the shorter arrow in Figure 1 projected onto the y-axis. The probability of a poor habitat is calculated as:

$$P\;\left(H_{poor}\right)={\left|\frac{e^{i\theta poor}}{\sqrt{2}}\right|}^{2}=\;\left(\frac{e^{i\theta poor}}{\sqrt{2}}\right)\bar{\;\left(\frac{e^{i\theta poor}}{\sqrt{2}}\right)}\;=\left(\frac{e^{i\theta poor}}{\sqrt{2}}\right)\;\left(\frac{e^{-i\theta poor}}{\sqrt{2}}\right)=e^{i\left(\theta poor-\theta poor\right)}\;{\left(\frac{1}{\sqrt{2}}\right)}^{2}=0.5$$

The solution of 0.5 here represents complete uncertainty, in the absence of any prior information, as to whether the bat habitat is good or poor, which is why the probability amplitudes are normalized by $\sqrt{2}$. That is, *P* (*H_good_*) = *P* (*H_poor_*) = 0.5, the same as in classical probability, because the phase of the amplitude specified by the shift of the wave function has not yet been specified from observations, which would also shift the value of the radical in the denominator. When the amplitude phase is determined, the probabilities of specific values of habitat conditions are then calculated using Born’s Rule:

$$P\;\left(H_{good}=v\right)\;=\langle P\;\left(H_{good}=v\right)\;\psi \;|\psi \rangle \;=\|P\;\left(H_{good}=v\right)\Psi {\|}^{2}$$

Born’s Rule tells how probable the possible outcomes are in a quantum system (see Supplementary Material). In quantum mechanics, this has been referred to as the collapse of the wave function [59,60], which is when a state changes probabilities or a new state emerges when the system is measured, the analog in BNs being when beliefs are updated in response to new priors.

Further note that, in quantum math, the sum of the squared magnitudes must equal 1:

$${\left|\frac{e^{i\theta good}}{\sqrt{2}}\right|}^{2}+\;{\left|\frac{e^{i\theta poor}}{\sqrt{2}}\right|}^{2}=1.0$$

**Figure 4:**
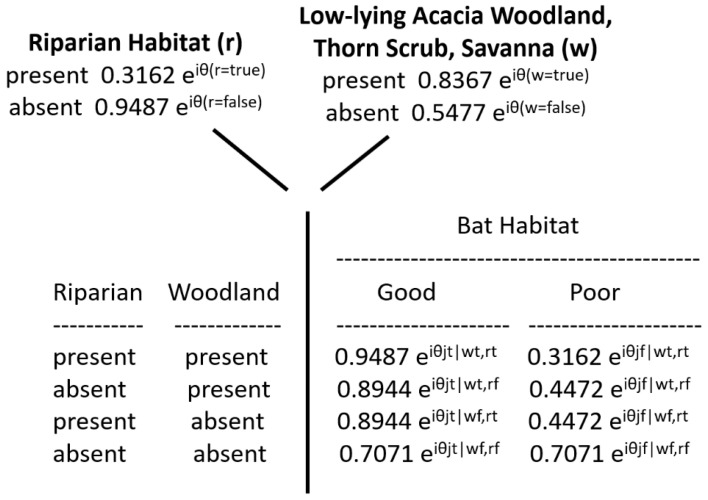
EcoQBN representation of the occurrence of two environments and their combinations to form quantum conditional probability values of the habitat quality for yellow-winged bats.

This is equivalent to the Law of Total Probability in Bayesian calculus, in which, among *i* states, $\underset{i}{\sum }\;\left(P_{i}\right)\;=\;1$. Thus, the full representation of the quantum conditional probabilities is denoted using the above equations, as shown in Figure 4. The prior probability values of the presence or absence of riparian habitat and the woodland, scrub, or savanna habitat are now each shown as the product of a constant scalar (being the square root of the classical conditional probability values), with factors using the phase of the amplitude (noted above). For example, the overall probability of a riparian habitat being present, as shown in the traditional Bayesian network (Figure 3), is 0.1; its square root is 0.3162, and the factor for the phase of the amplitude is denoted as $e^{i\theta \left(r=true\right)}$. In a similar way, the quantum conditional probability values are also shown using the same kind of square-root converted scalar, but with the phase of the probability amplitudes which include combinations of the conditions of the two kinds of habitats. This specification constitutes the general solution space for the EcoQBN representation. Specific solutions depend on the base probabilities (Figure 3), and on the amplitude phases which pertain to the conjunctions of the values of the two habitats. As additional habitat categories and other resource-selection sequences are added, the EcoQBN for yellow-winged bat habitats would be far more efficient than a traditional BN, which would require many additional nodes, links, and probability values.

#### 3.2.2. Example 2: Boreal Forest Development under Disturbance Regimes

**Figure 5:**
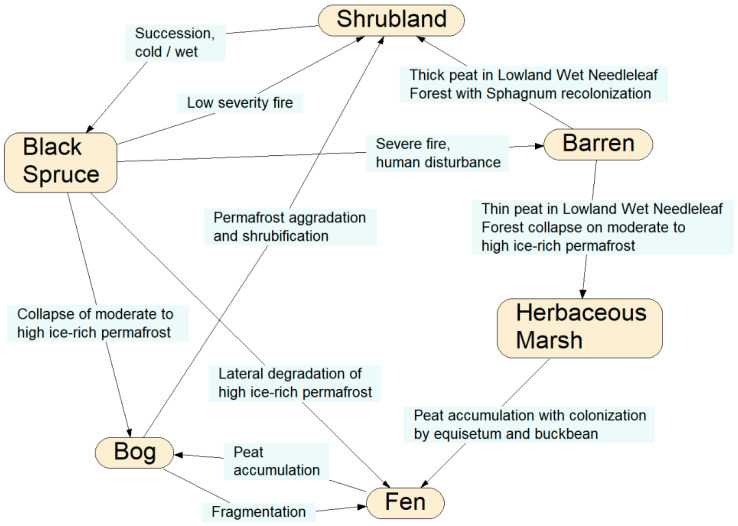
Example of a state-transition network of terrestrial ecological communities in central boreal Alaska, highlighting state transitions specific to black spruce (*Picea mariana*) forests. The nodes represent specific vegetation and land cover types, and the links between the nodes are potential developmental pathways showing specific disturbance conditions causing each potential transition [Source: after M. T. Jorgenson and H. Genet, pers. comm.].

This is a framework for an example of the representation of an intransitive ecological system. Northern hemisphere mid-boreal forests are under increasing stress from a variety of anthropogenic and natural disturbances, including wildfires, regional warming, increased precipitation, permafrost thaw, and population cycles of grazing animals such as hares, lemmings, caribou, and muskoxen, each having different impacts on vegetation conditions. Sequences of disturbance events can result in different state-transition changes of a vegetation type in a particular physiographic setting (Figure 5).

For example, consider a mature black spruce forest on a steep upper slope, and these two particular temporal sequences of disturbance events: (1) regional warming with increased mean daily temperature, fire, and precipitation; and (2) increase precipitation, fire, and populations of grazing animals. Sequence (1) could lead to the site becoming tinder-dry, then incurring high-intensity crown and understory fire, followed by mass wasting and slope failure from the rainfall runoff, resulting in a scarified slope of bare soil or bedrock, so that the original black spruce forest transitions into a post-fire barrens. Sequence (2), however, could lead to the site first becoming verdant and rich with the ground-level growth of grasses and forbs, then a low-intensity cool ground fire which could stimulate more vegetation growth, which then provides prime forage for grazing animals, restoring the site to black spruce forest.

**Figure 6:**
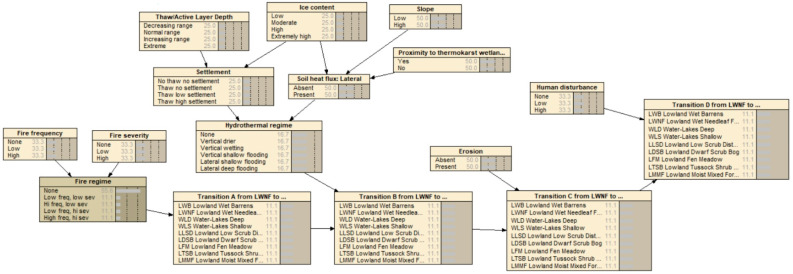
Example of a partial specification of a traditional BN representation of the black spruce and other boreal forest transitions shown in Figure 5.

In all, at least five distinct transition pathways exist under various disturbance regimes whereby the black spruce forest can eventually return to black spruce forest (Figure 5). In order to model all of the possible state transition changes under a realistically wide variety of (sequences of) disturbances, stressors, and environmental conditions would entail a massive decision tree of all of the possible combinations and outcomes, and thus a massive time-expanded DBN model with many latent variables and linkages depicting all possible sequences and permutations of disturbances, stressors, and environmental conditions (Figure 6).

Instead, the BN model could be made most terse and efficient by using quantum probability amplitudes. Such a fully-specified EcoQBN would account for all of the transition probabilities represented as wave functions, and intransitive changes back to black spruce forest. Future uncertainties of climate change can be further represented as a superpositioning of states; the prediction of future outcomes in systems with feedback cycles under uncertainty can lead to superpositioning, which is when systems—future ecotype transitions—can exist simultaneously in >1 dominant quantum probability state. The EcoQBN would also represent the noncommutativity of various temporal sequences of the disturbance events.

**Figure 7:**
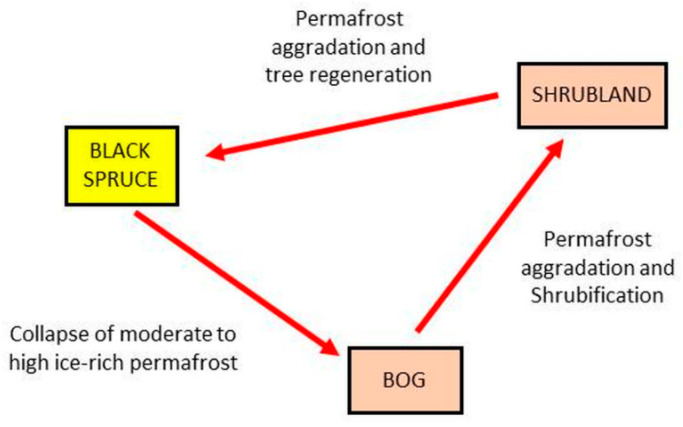
One pathway for the black spruce transitions (from Figure 5). The nodes are vegetation and land cover types, and the links depict potential transitions between types, labeled by the types of disturbance events causing each transition.

In a simple depiction, consider one subset of the full transition network (Figure 7) in which black spruce forest can change into a bog under the thaw and collapse (thermokarst) of permafrost; the bog can become shrubland under the subsequent aggradation of permafrost and peat with conversion from moss-lichen or herb-grass conditions to shrub-dominated conditions; the shrubland can again become black spruce forest under further permafrost aggradation and tree regeneration. This simple three-state process now represents an intransitive network, as with roshambo, and can be modeled using the quantum state-transition formulae presented earlier. However, this figure contains a cycle, so it cannot per se form a BN, as this violates the requirement that BNs must be acyclic, unless they are self-replicated.

Furthermore, higher-order spruce forest circuits also can be identified from Figure 5, such as transitions of black spruce forest to barrens, then herbaceous marsh, fen, bog, shrubland, and back to spruce forest. An EcoQBN representation would use the path trajectory calculations presented further above to account for the probabilities of the spruce forest resulting from all possible pathways simultaneously, as in a multi-dimensional Hilbert stage-space. As with the roshambo example, no prediction or diagnosis can be made with a traditional BN representation of such state-transition dynamics unless >1 state is specified, whereas an EcoQBN representation can depict all of the states simultaneously with a superposition function which further resolves to individual state probabilities as information is gathered and actual changes are hypothesized or actually occur.

#### 3.2.3. Example 3: Signal Entrainment in Faunal Species

A final example pertains to quantum wave-function analogues to signal entrainment in birds and insects. Many animal species give aural or visual signals which serve as different kinds of conspecific cues. In some species, signals from the reciprocating organism can become entrained in some way to the signal-giver. Entrainment refers to when the second organism’s signals are given at the same rate as the first’s, and synchrony refers to entrainment when both signals are given at the same instant. Examples of signal entrainment include the synchrony of bird songs or calls, and examples of signal synchrony include the visual synchrony of firefly flashes, and the behavioral synchrony of the stylized mating movements of albatrosses and grebes.

**Figure 8:**
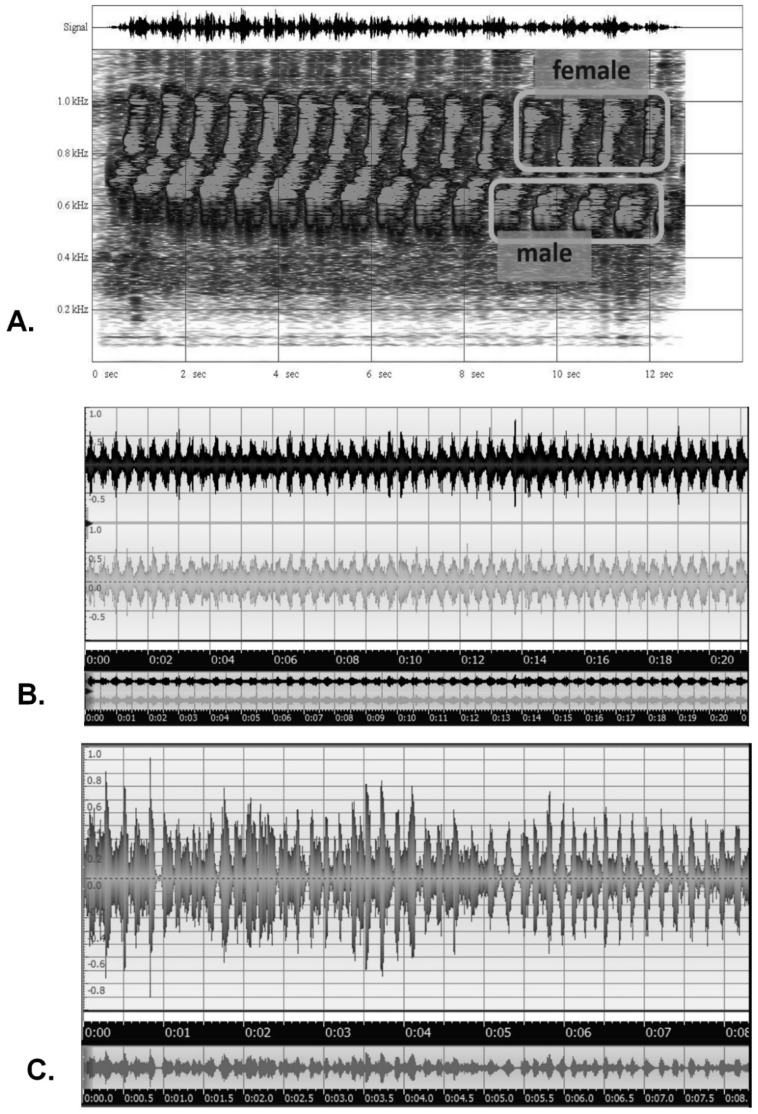
Examples of signal entrainments in ecology: a sound spectrogram (frequency vs. time plot) of the duetting of male and female marbled wood quail in the Upper Amazon of Ecuador (**A**); an amplitude vs. time plot of the synchronization of multiple chirping field crickets in Western U.S.A. (**B**); an amplitude vs. time plot of the eventual entrainment and synchronization of chirp calls in a colony of Vaux’s swifts in Oregon, U.S.A. (**C**) [Recordings by Bruce G. Marcot].

Entrainment without synchrony occurs as song duetting in birds, specifically what is termed ‘antiphonal singing’, in which the male and female are so tightly coordinated that they sound as one, as is most commonly heard in tropical birds. An example is the marbled wood quail (*Odontophorus gujanensis*), shown in a time-based sound spectrogram in Figure 8A, in which the male’s lower-frequency notes are immediately followed by the female’s response notes in a rapid, entrained fashion, even as the entire series slows. An example of entrainment with synchrony is the chirping calls of field crickets (*Gryllus* sp.), which attain unison (Figure 8B). Another novel example is the chirping calls of Vaux’s swifts (*Chaetura vauxi*) within a colony (Figure 8C); in this case, the chatter begins rather randomly, but soon attains a sustained entrainment and synchrony, perhaps serving some group behavior or colony-recognition function. In general, signals showing entrainment with synchrony generally conform to the achievement of a sort of amplified mutual resonance, such as with multiple metronomes on a balanced platform [61], walking on a suspension bridge [62], and many other cases [56,63,64].

Entrainment with and without synchrony between two organisms or species (1 and 2) can be generally modeled with simultaneous equations:

$$\frac{d\theta_{1}}{dt}=w_{1}+A_{2}sin\left(\theta_{2}-\theta_{1}\right)$$

$$\frac{d\theta_{2}}{dt}=w_{2}+A_{1}sin\left(\theta_{1}-\theta_{2}\right)$$

in which entrainment occurs when *A*_2_ *= A*_1_, and synchrony is defined when *w*_1_ = *w*_2_. These equations describe overlapping signal waveforms given between two organisms or species, such as between duetting birds of a pair, between predator–prey interactions, and other potential ecological situations. An EcoQBN can use these equations to calculate the posterior probabilities of signal entrainment, as an analogue to quantum wave-form interference and resolution of the indeterminate wave functions to a single dominant frequency. In quantum information processing, waveform interference can be modeled mathematically using rules of complex numbers, and can usefully describe potentially conflicting uncertainty among alternative states [14,22]. When the uncertainty becomes reduced or resolved, the quantum interference term, represented as cos (*ϴ*), becomes trivial, such that the wave function resolves to the classical Bayesian representation, in this case, probability functions denoting coordinated rhythmic patterns. In an EcoQBN, waveform interference and signal entrainment can also represent ecological tension and contrary forces, such as with species competition, coevolution in predator–prey systems, a fundamental niche space collapsing to realized niche space, and many other situations in which stochastic dynamics or uncertainty are prevalent.

## 4. Discussion

### 4.1. Characteristics of EcoQBNs

EcoQBN models can represent conditions of high interest in ecological research which are not amenable to traditional Bayesian statistical and network probability modeling. An EcoQBN modeling framework presents the capacity for the representation of several unique system characteristics, particularly with intransitive or hyperintransitive ecological systems in which all possible outcomes (transitions to other states) are equally probable until >1 prior condition is specified. In a traditional BN formulation, knowing the state of a system, which may have resulted from >1 causal pathway, may be inadequate information from which to deduce its cause, or to predict its future. In a BN, diagnosing potential causes entails tracing backwards in the state-transition table from a given state to its multiple potential progenitors, whereas an EcoQBN can represent all of the potential causes in one superposition function. Furthermore, in traditional modeling, it may not be possible to predict changes to an ecosystem even by knowing a specific set of affectors (e.g., climate change, anthropogenic disturbances, environmental stressors) without knowing their specific sequence, whereas EcoQBNs can account for systems with such noncommutative properties. Because of these and other properties discussed in this paper, EcoQBNs seem well suited for depicting and modeling situations of ecological stress, biological conflict, and the emergence of multiple dominant conditions, such as those found in evolution and adaptation, species competition, symbiosis and mutualism, polymorphic populations, and much more.

EcoQBNs also can represent non-Markovian, or higher-order Markovian, effects, in which the probability of some event depends not only on the immediate parent(s) of that event, but also to previous generations of that parent (deeper layers of the network) or even to entirely different arms of the network (entanglement). This can usefully represent systems with ‘ecological inertia’ [65] resulting from time lag effects, such as from regional climate change, secondary succession from soil seed banks, cumulative historic stressors, autocorrelation with >1 previous time period, and with non-Markovian, multi-state transitions (e.g., Reference [66]). In general, the EcoQBN framework can usefully represent a new view of the intricacy of ecosystems with multiple, complex correlations, conditionalizations, joint distributions, and the mutual conditioning of environments and organisms.

### 4.2. Limitations of the EcoQBN Approach

For useful, practical application, EcoQBNs need to be structured and parameterized using empirical data, expert knowledge, or a combination, much as traditional BNs can be devised. The network structure could be developed using existing methods of machine-learning algorithms and expert elicitation in order to identify node configurations best representing correlational, causal, or logical relationships, but such approaches could miss important relations representing feedbacks, time lags, and sequences of influences.

However, because of the attributes of entanglement which will be represented in QBNs, it is less key to identify specific correlational links among input nodes, as is needed in traditional BNs. The parameterization of variables, including the identification of appropriate states and, depending on their variable type, their categories, ordinal levels, continuous-value ranges, or functions, also entails the identification of the forms of the their wave functions and interference effects which depend on degrees of uncertainty. These may be difficult to empirically quantify in quantum-math structures. Further work is likely needed to develop methods of learning algorithms for variable parameterizations in EcoQBNs, which could spark new ways of viewing and researching ecological systems.

QBNs represented by a Hilbert two-axis graphic are essentially two-state systems, and would require higher dimensionality for nodes with >2 states. The quantum math becomes increasingly more complex with >2 states and >2 causal nodes, but the framework is available (e.g., see Reference [22] for an example of an n-state system).

### 4.3. Toward Operational Computing

Solving QBNs, and by extension EcoQBNs, entails developing and solving mathematical representations by hand, or perhaps by using coding math-based programming languages and computing systems. At present, there seems to be no modeling shell or computing program, commercial or open-source, by which QBNs can be constructed and run. The development of such shells would greatly aid the testing, further development, and adoption of this approach in ecological modeling, opening entirely new avenues of conceptualizing, monitoring, and perhaps managing ecological systems and natural resources. Eventually, EcoQBN modeling will also benefit from advances in quantum computing (e.g., References [67,68]), including the development of new machine-learning algorithms for structuring and parameterizing models [69,70], and spurring new approaches to their calibration and validation. There may be potential applications of quantum Markov chain theory [17] in developing such new modeling tools.

## 5. Summary and Conclusions

Many current ecological analysis structures—such as state-and-transition models, individual-based movement simulations, and ecological disturbance models—could greatly benefit from an EcoQBN approach. The framework can help structure and analyze intransitive and noncommutative ecological systems in which traditional BN solutions are infeasible or, at best, would require highly complex latent variable structures, especially when uncertainty is very high.

QBNs use superpositioning to represent the probabilities of being in >1 state at a time, which is similar to how classical Bayesian calculus provides normalized probabilities of multiple states. The difference is that when a QBN model is run multiple times using the same prior probability inputs, the resulting output state probabilities can vary according to the superposition probabilities and waveform interference properties of the various states denoted by the quantum amplification formulae. Furthermore, a QBN can have multiple forms of the output variable, representing different initial conditions, difference sequences in which the inputs are specified, different interference patterns of input variables (priors), and other non-traditional BN modeling assumptions. As such, in this way, a QBN can produce various outcomes for one set of initial inputs, dependent on the hidden elements (latent variables) of inherent stochasticity in the causal network.

In summary, the characteristics of problems which can be solved with EcoQBNs which cannot easily, or at all, be solved with traditional BNs include:

- problems with noncommutative properties of dependencies, in which the outcome depends on the sequence of introducing elements into some interaction, such as with disturbance events and the order of specific management activities affecting conditions of ecological communities;
- problems with the intransitive properties of dependencies, in which there is no fixed, linear hierarchy of the relationships among multiple parameters, such as with many conditions of species interactions and the coevolution of adaptive traits;
- problems with complex positive or negative feedback loops which increase or depress the amplitudes of responses, such as with accelerating regional warming from decreased albedo or predator–prey cycles, respectively; and
- problems of variable entanglement, in which the input variables can affect each other even if they are not causally linked, such as with hidden stressors and latent variables, and in which cause and effect can reverse (mutual causality), such as with interference competition.
- In general, QBNs, including their ecological applications, can address:
- problems in which the dominant probability outcomes are nonintuitive and defy traditional probability calculus;
- problems in which there can be >1 dominant quantum probability outcome, each *P* > 0.5 (superpositioning);
- problems of systems with chaotic behavior, particularly to recognize the tipping points and thresholds of major system shifts;
- problems in which the basic causal structure and parameters are fixed, but different trials (model runs), based on the same inputs, can yield different outcomes, and in which one set of the initial inputs can produce various outcomes, providing a new means of depicting and modeling stochastic systems and the implications of various degrees of knowledge uncertainty;
- problems in which outcomes are nonintuitive and defy traditional probability calculus.

The EcoQBN approach is a new way of thinking of the dynamics of ecological systems beyond mechanistic modeling, as it holds great promise for ecological description and prediction in many topic areas. It can inspire new ways of conducting research on ecological systems, such as to study non-hierarchical interactions, feedback relationships, and multiple and interchangeable causality. It is offered here as a nascent field needing the further application of the quantum mathematical structures, new learning algorithms for network design and parameterization, and, eventually, adjuncts to existing modeling shells or entirely new software programs to facilitate model development, application, and validation.

## Figures and Tables

**Table 1:** Comparison of the attributes of classical probability theory, such as that which is used with Bayesian statistics and networks, with quantum probability theory, such as that which is used with quantum Bayesian networks.

Classical Probability Theory	Quantum Probability Theory
Variables are independent and do not influence each other, if they are not linked and do not share a common parent node.	Variables can be entangled and dependent (mutually influencing), even if they are not linked and do not share a common parent node.
States of variables are mutually exclusive.	States of variables are not mutually exclusive.
States are represented by discrete probability values that can be resolved with observations.	States are represented by a probability wave function and can have interference patterns that resolve with observations.
There can be only one dominant-probability state with *P* > 0.5.	There can be >1 dominant-probability quantum amplitude, each with *P* > 0.5.
Classical probabilities of states sum to 1.	Probabilities of quantum superposition states can sum to >1.
Probabilities of states are represented by classical probability calculus.	Probabilities of states are represented by probability amplitudes of the wave function.

## Data Availability

Data is contained within the article or supplementary material.

## Supplementary Materials

The following are available online at https://www.mdpi.com/article/10.3390/e23040441/s1, S1 Calculations of probabilities of states from a Hilbert space projection; S2 Calculations of conditional probabilities of conditions for the bat habitat example discussed in the text; S3 Calculations of prior probabilities of conditions for the bat habitat example disussed in the text.
