# Supplementary material for: EcoQBNs: First Application of Ecological Modeling with Quantum Bayesian Networks
Source: Entropy (Basel). 2021 Apr 9;23(4):441. doi: 10.3390/e23040441 (PMC8069849; doi:10.3390/e23040441)
Supplement: Supplementary file 1 [file entropy-23-00441-s001.pdf]

Supplementary Material

for: Marcot, B. G. EcoQBNs: First application of ecological modeling with quantum Bayesian networks. *Entropy*

S1 Calculations of probabilities of states from a Hilbert space projection

This example assumes a two-state system, such as whether habitat *H* for a species is good or poor. The Hilbert space describes a vector of unit length on a 2-axis representation of the two habitat states *H1* and *H2* (e.g., text Fig. X1). This system state vector *S* makes up all state events, and is a probability function that maps state events into probability values [0,1].

The x and y axes of the Hilbert space representation are represented by two column vectors:

$$X = H1 = \begin{bmatrix} 0 \\ 1 \end{bmatrix}, \qquad Y = H2 = \begin{bmatrix} 1 \\ 0 \end{bmatrix}$$

and the superposition state *S* -- representing each state simultaneously -- is represented by:

$$S = \frac{e^{i\theta x}}{\sqrt{2}} H1 + \frac{e^{i\theta y}}{\sqrt{2}} H2$$

where

$$\frac{e^{i\theta}}{\sqrt{2}}$$

defines the probability amplitudes (wave amplitudes, described by complex numbers; see text) and $e^{i\theta}$ = the phase of the amplitude (a "shift" of the waveform).

As noted in the text, the probability amplitude squared is the classical state probability value, which is calculated by multiplying the amplitude by its complex conjugate. In the Hilbert state space representation, for habitat state *H2* (text Fig. 1), this is calculated as:

$$P(H2) = \left| \frac{e^{i\theta y}}{\sqrt{2}} \right|^2 = \left(\frac{e^{i\theta y}}{\sqrt{2}} \right) \left(\overline{\frac{e^{i\theta y}}{\sqrt{2}}} \right)$$

$$= \frac{e^{i\theta y}}{\sqrt{2}} \cdot \frac{e^{-i\theta y}}{\sqrt{2}} = e^{i(\theta y - \theta y)} \left(\frac{1}{\sqrt{2}} \right)^2 = 0.5$$

As noted in the text, the solution of $P(H2) = 0.5$ denotes complete uncertainty in this two-state system because the value of the wave form shift parameter Θ has not been specified, which would otherwise denote the degree to which the two state wave functions differ and would potentially result in non-uniform probability outcomes.

S2 Calculations of conditional probabilities of conditions for the bat habitat example discussed in the text, based on a non-ecological example given in Trueblood et al. (2015).

With:

r = riparian habitat, with condition *i*,
w = woodland, scrub, or savanna habitat, with condition *j*, and
H = bat habitat condition, with conditions *good* or *poor*,

joint probabilities are determined from the conditional probability table (text Fig. X2) as:

$$P(H_{good}|r_i, w_j) \cdot P(r_i) \cdot P(w_j)$$

Similarly, in the quantum Bayesian network (QBN), let bat habitat, riparian, and woodland habitat conditions be associated with projectors *P*, *Q*, and *S*, respectively. Then their joint probabilities can be described by use of Born's Rule describing the absolute magnitudes of the vectors:

$$\left\|Pw_{good}\psi r_{i,}w_j\right\|^2 \; \|Qr_i\psi\|^2 \; \left\|Sw_j\psi\right\|^2$$

where the conditional state is given by:

$$\psi_{r_i,w_j} = \frac{Sw_jQr_i\psi}{\left\|Sw_jQr_i\psi\right\|}$$

If the projectors *Q* and *S* do not commute, then the conditional state will depend on the *order* in which these two variables are specified, so that

$$\psi_{r_i,w_j} \neq \psi_{w_j,r_i}$$

which further denotes that

$$P(H_{good}|r_i, w_j) \neq P(H_{good}|w_j, r_i)$$

This is an ecological noncommutative condition, for example, if the bat were to first seek the overall woodland condition and then within that seek the riparian condition, and if the probability (frequency) of using that site was different than if it first followed riparian areas to where it would embed within a woodland condition. By the ecological principle of hierarchical habitat selection, the former is more likely the case, where the bat would seek the woodland condition that is more generally available across the landscape, and then within that seek specific and more constrained riparian conditions. (However, for this species, such habitat selection behavior is speculative and intended here for demonstration of the statistical modeling approach.)

From the above framework, in a QBN, the probability values in the traditional conditional probability table (CPT; text Fig. 2) are replaced by probability amplitudes, as detailed in the main text. Particularly if the system being described is ecologically noncommutative such as described above, then the resulting CPT can be referred to as conditional quantum probability table or CQPT, and different CQPT values would need to be calculated for different sequences with which the input variables (habitat selection sequences) are specified. Note that noncommutative conditions -- the sequence in which prior conditions are specified -- essentially violate local Markov conditions and are thus not solvable with traditional Bayesian calculus without introducing additional complexity with latent variables that specify the sequences and their unique outcomes, whereas a QBN can inherently deal with such conditions.

Reference

Trueblood, J.S.; Mistry, P.K.; Pothos, E.M. A quantum Bayes net approach to causal reasoning. In *Advanced Series on Mathematical Psychology. Volume 6. Contextuality from Quantum Physics to Psychology*; Dzhafarov, E., Jordan, S., Zhang, R., Cervantes, V., Eds.; World Scientific: Singapore, 2016; pp. 449–464.

S3 Calculations of prior probabilities of conditions for the bat habitat example discussed in the text and for the conditional quantum probability table discussed in Supplement 2.

In the CQPT, the prior probabilities are derived as follows. First, presume that only 10% of a given landscape consists of riparian areas, which is the basis for the value 0.1 in the CPT of text Figure 3, or

$$P(r_{present}) = 0.1$$

Using Born's rule (see text) in the CQPT, this prior probability converts to:

$$P(r_{present}) = \left\| \sqrt{0.1} e^{i\theta(r_{present})} \right\|^2$$

$$= \left(0.3162 e^{i\theta(r_{present})}\right)\overline{\left(0.3162 e^{i\theta(r_{present})}\right)}$$

$$= \left(0.3162 e^{i\theta(r_{present})}\right)\left(0.3162 e^{-i\theta(r_{present})}\right)$$

$$= (0.3162)^2 e^{i[\theta(r_{present})-\theta(r_{present})]}$$

$$= 0.1$$

because

$$e^{i[\theta(r_{present})-\theta(r_{present})]} = e^{i\cdot 0} = e^0 = 1$$

and where $\overline{\left(0.3162 e^{i\theta(r_{present})}\right)}$ = the conjugate matrix.
